# Geographic isolation and climatic variability contribute to genetic differentiation in fragmented populations of the long-lived subalpine conifer *Pinus cembra* L. in the western Alps

**DOI:** 10.1186/s12862-019-1510-4

**Published:** 2019-10-17

**Authors:** Endre Gy Tóth, Francine Tremblay, Johann M. Housset, Yves Bergeron, Christopher Carcaillet

**Affiliations:** 1 Forest Research Institute (IRF), University of Quebec in Abitibi-Témiscamingue (UQAT), 445 Boul. de l’Université, Rouyn-Noranda, QC J9X 5E4 Canada; 2 National Agricultural Research and Innovation Center (NARIC), Forest Research Institute (FRI), Várkerület u. 30/A, Sárvár, 9600 Hungary; 3 Centre for Forest Research (CEF), University of Québec in Montréal (UQAM), C.P. 8888, succ. Centre-ville, Montréal, QC H3C 3P8 Canada; 4 Ecole Pratique des Hautes Etudes (EPHE), Paris Sciences & Lettres University (PSL), Paris, France; 5 Alcina, 10 rue des Amaryllis, 34070 Montpellier, France; 6 Laboratory for Ecology of Natural and Anthropised Hydrosystems (UMR 5023 CNRS UCBL ENTPE), Université Lyon 1, Villeurbanne Cedex, France

**Keywords:** Diversity, Differentiation, Climatic variability, Gene flow, Isolation, *Pinus cembra*

## Abstract

**Background:**

Genetic processes shape the modern-day distribution of genetic variation within and between populations and can provide important insights into the underlying mechanisms of evolution. The resulting genetic variation is often unequally partitioned within species’ distribution range and especially large differences can manifest at the range limit, where population fragmentation and isolation play a crucial role in species survival. Despite several molecular studies investigating the genetic diversity and differentiation of European Alpine mountain forests, the climatic and demographic constrains which influence the genetic processes are often unknown. Here, we apply non-coding microsatellite markers to evaluate the sporadic peripheral and continuous populations of cembra pine (*Pinus cembra* L.), a long-lived conifer species that inhabits the subalpine treeline ecotone in the western Alps to investigate how the genetic processes contribute to the modern-day spatial distribution. Moreover, we corroborate our findings with paleoecological records, micro and macro-remains, to infer the species’ possible glacial refugia and expansion scenarios.

**Results:**

Four genetically distinct groups were identified, with Bayesian and *F*_ST_ based approaches, across the range of the species, situated in the northern, inner and south-western Alps. We found that genetic differentiation is substantially higher in marginal populations than at the center of the range, and marginal stands are characterized by geographic and genetic isolation due to spatial segregation and restricted gene flow. Moreover, multiple matrix regression approaches revealed effects of climatic heterogeneity in species’ spatial genetic pattern. Also, population stability tests indicated that all populations had experienced a severe historical bottleneck, no heterozygosity excess was detected, suggesting that more recently population sizes have remained relatively stable.

**Conclusions:**

Our study demonstrated that cembra pine might have survived in multiple glacial refugia and subsequently recolonized the Alps by different routes. Modern-day marginal populations, at the edge of the species’ range, could maintain stable sizes over long periods without inbreeding depression and preserve high amounts of genetic variation. Moreover, our analyses indicate that climatic variability has played a major role in shaping differentiation, in addition to past historical events such as migration and demographic changes.

## Background

Amounts and distributions of genetic variation among populations and across species’ ranges are results of complex interplay of gene flow, genetic drift and natural selection [1]. Geographic and environmental factors may also contribute to these processes and further influence species’ modern-day patterns of genetic diversity [2]. Genetic variation is often unequally partitioned across a species’ natural range and can differ between populations at the geographical center and margins of the range [1, 3]. Generally, high gene flow tends to occur in central populations, leading to genetic homogenization, while populations at the range margin experience low gene flow, strong genetic drift and different selection regimes, which often results in increased genetic differentiation among populations [4, 5]. Genetic differentiation develops slowly as substantial differences in genetic variation, such as changes in allele frequencies, gradually accumulate among populations or geographical regions. Particularly high genetic differentiation can manifest at the margins of a species’ range, where population fragmentation and isolation are more likely to influence genetic processes [6, 7].

Fragmentation segregates a continuously distributed population into smaller, spatially isolated habitats, leading to differences in the genetic architecture within and between the resulting populations [8]. Numerous factors drive range fragmentation, including climate-driven dynamics that constantly affect a species’ natural distribution [3]. Moreover, human-induced habitat modifications such as deforestation can cause rapid changes in the distribution of a species, leading to population size reduction, fragmentation and spatial segregation [9, 10]. Consequently, populations became geographically and genetically isolated.

Isolation, together with reduction in habitat quality and small population size, is predicted to result in the decline, and ultimately extirpation, of a population [8, 9]. Usually, isolation can increase the chance of random genetic drift, raise inbreeding rates, reduce interpopulation gene flow, and increase the probability of local extinction [11]. If this isolation is temporary, we expect to see a loss of heterozygosity together with a reduction in individual fitness, while long-term isolation can cause a population bottleneck that reduces allelic variation and ultimately limits the ability of a species to respond to selection [8, 12].

Fragmented and isolated populations, especially those at the margin of a species’ range, often inhabit suboptimal, heterogeneous environments [13, 14]. Fluctuating environmental conditions, including variation in the local or regional climate, can induce severe stress that can considerably alter the genetic variation of a species through divergent selection processes [15], and may lead to increased genetic differentiation between populations on a spatial scale [16]. Environmental variability (or heterogeneity) has long been recognized as an important driver of genetic variation and differentiation [17, 18], and several studies have also shown statistical associations between neutral genetic variation and environmental heterogeneity [19–22]. The latter correlations may be interpreted as evidence of diversifying selection acting over whole genomes, including on putatively neutral loci, and allows us to infer the long-term effects of environmental conditions on genetic variation [19, 23].

**Fig. 1:**
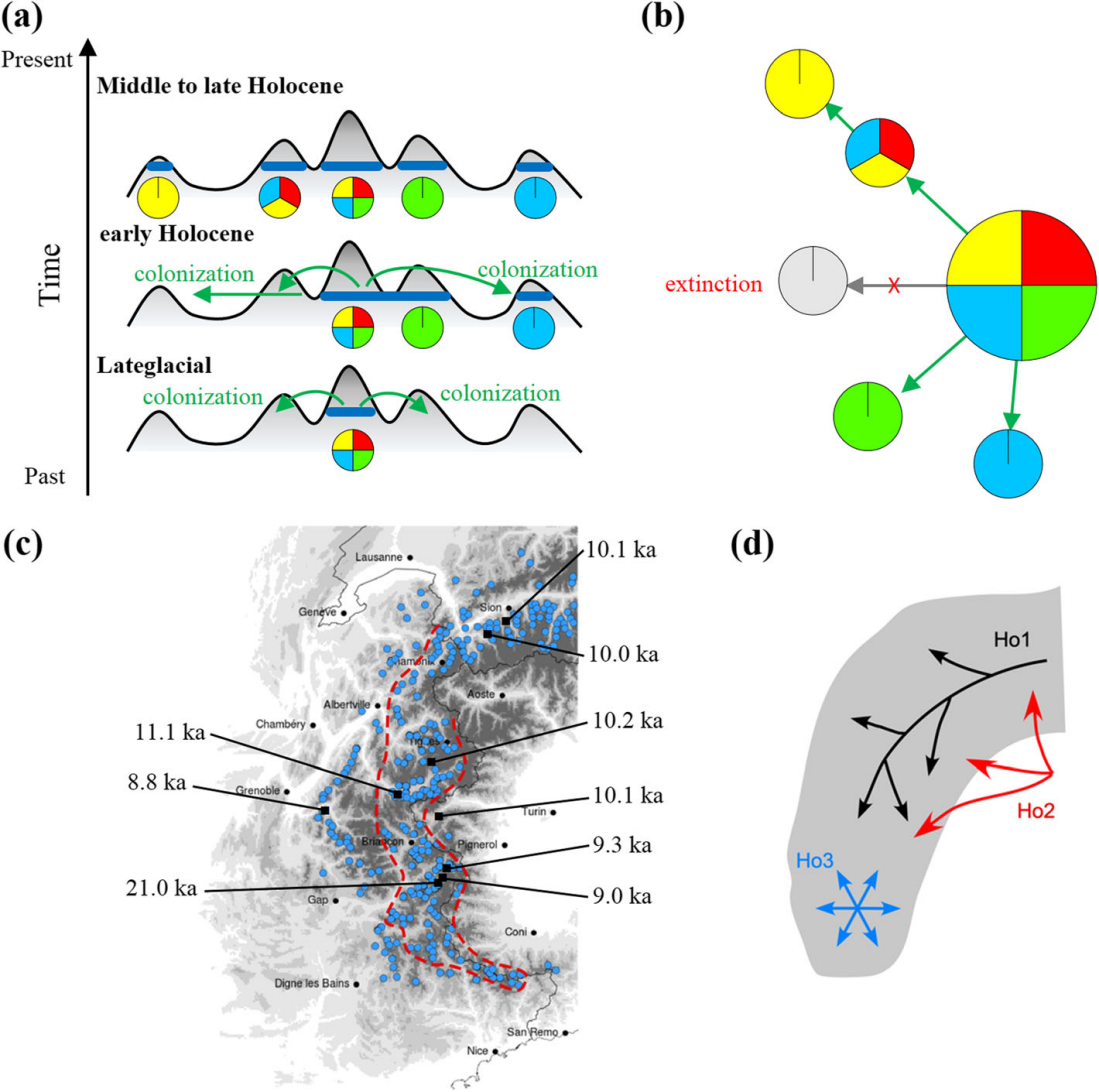
Hypothetical scenarios of postglacial expansion in the extreme western part of the range of *Pinus cembra*. **a** ‘Classic’ temporal scenario of colonization and extinction, based on glacial refugia in the Carpathians or the eastern Alps, and migration through central massifs/valleys with four hypothetical haplotypes (number chosen for the conceptual exercise) illustrating colonization, expansion and extinction processes. **b** Modern spatial pattern of the hypothetical haplotypes, their main/central (large polygon) and peripheral/fragmented populations (small polygons), their migration routes (arrows), and their eventual extinction or absence of immigration (grey area). **c** Actual locations of main *P. cembra* forests in the western Alps (the red dashed line distinguishes main central populations from peripheral/fragmented populations), and locations of first dated supporting subfossils (see Additional file 1). **d** Schematic illustration of the three hypothetical scenarios explaining the species’ distribution in the western Alps: the ‘Classic’ scenario (Ho1); ‘Southeast Alpine refugia’ scenario (Ho2, based on [25, 26]; and ‘Intra-Alpine southern refugia’ (Ho3 based on [27]). The three scenarios are not exclusive but complementary

Cembra pine (*Pinus cembra* L.) provides an interesting model to measure genetic variation within its distribution area and investigate the drivers that contribute to genetic differentiation within and among populations. This tree is a keystone species which grows up to the treeline in high mountains from the western Alps in western Europe to the Carpathians in eastern Europe. Historically, the range of this species also encompassed the Euro-Asian boreal forests from eastern Siberia to the western Urals [24]. Because of its restriction to the cold-snowy, subalpine forest belt in the Alps, its current distribution area is naturally fragmented by alpine tundra, rocky summits or glaciers at high elevations, or deep alpine valleys. While *P. cembra* dominates forests in central valleys and massifs of the Alps, where the continental-type climate is optimal for its growth, the climate of the peripheral mountain massifs varies from oceanic in the north to Mediterranean in the south. Populations in these peripheral areas become increasingly sporadic and sparse and, ultimately, absent. *Pinus cembra* is a long-lived species (up to 500–1000 years) with long generation intervals, which should intuitively limit its capacity for genetic differentiation during a short period like the interglacial Holocene. However, other properties may promote genetic differentiation. For example, *P. cembra* has heavy seeds and strict zoochory, which prevents long-distance seed dispersal and hinders its colonization potential, but this also enhances the founder effect of initial colonizers. In practice this may have caused a bottleneck effect in isolated peripheral mountains when they were first colonized during the postglacial period (Lateglacial or early Holocene), ultimately resulting in the reduced genetic diversity that is believed to exist in the present day peripheral populations (Fig. 1a–b, Additional file 1: Table S1–S2). Finally, human-induced changes in land use over the last millennium may also have altered the genetic diversity of populations, most notably in peripheral populations characterized by low cover or tree density. Detailed information on this species’ fossil evidences (micro and macrofossil records) can be found in the supplementary material (Additional file 1).

Previous molecular genetic studies of cembra pine populations have revealed substantially higher differentiation and genetic diversity in populations from the Carpathians compared to those in the Alps. Generally, studies on Alpine populations observed low to moderate levels of genetic diversity and low genetic differentiation [28, 29]. This gradual decrease in genetic diversity from east to west could be explained by postglacial recolonization from eastern European glacial refugia, and the associated genetic drift (due to founder events or bottlenecks) and corresponding reductions in gene flow that have ultimately shaped the present-day genetic variation [25]. However, marginal populations in the northern Alps exhibit significantly lower genetic diversity and higher genetic differentiation than populations in the central Swiss Alps; features indicative of genetic structuring within the main central range and possible existence of other glacial refugia in the southeastern Alps [25]. These indications have been supported by pollen evidence and, even better, plant macroremains from the Italian plains and foothills [26, 30, 31]. Most studies agree that fragmented *P. cembra* populations are predominantly characterized by weak gene flow, genetic drift and high genetic differentiation [28, 29]. *P. cembra* populations located in the main range in the central Alps are sufficiently well-connected to allow recurrent mixing through gene flow [28]. However, towards the range margin, fragmented populations become increasingly segregated and isolated with limited genetic mixing. Isolated stands of cembra pine are often inbred, which is presumably caused by self-fertilization and mating between related trees. This results in strong differentiation even between geographically close populations [32–34].

The intraspecific genetic variability and structure of cembra pine populations in the central Alps and the Carpathians have been previously investigated, but not the genetic structure within the western Alps, which encompasses naturally fragmented forests (Fig. 1c). We hypothesized that imprints of continental-scale genetic processes will be preserved at the regional scale, and manifested in a pattern of decreasing genetic diversity from the central *P. cembra* populations, growing in an optimal climate, to peripheral populations in isolated mountain massifs characterized by low tree density or cover. Although the genetic pattern in the western Alps is currently unknown, paleoecological information has prompted proposals that ‘nunataks’ (unglaciated mountain summits surrounded by ice) may have preserved boreal-type habitats, and thus provided previously unsuspected glacial refugia for *P. cembra*, in the southwestern Alps [35]. Therefore, we aimed to: (i) explore the regional genetic diversity of this species and analyze its partitioning between populations inhabiting the central range of the western Alps and the isolated populations at the distribution margin, (ii) investigate genetic differentiation and structure between different geographic regions, and (iii) evaluate effects of geographic isolation and environmental heterogeneity on the species’ spatial genetic pattern, taking into account the different refugia and expansion scenarios (Fig. 1d).

## Results

Mature cembra pine (*Pinus cembra*) trees (727), from 22 populations, were genotyped with seven nuclear microsatellite markers (nSSR) from western range of the species’ in the French and Italian Alps (Table 1). Total proportion of missing data among the genotyped samples (failed genotyping) was 2.8%, more or less evenly distributed among individuals and loci. Percentages of polymorphic loci in all populations were 100%. Overall, 95 alleles were detected and the number of alleles between loci ranged from 5 (Pc18) to 24 (Pc23). Population-specific (private) alleles were found in 11 populations and distributed among 5 loci (Pc5, 7, 22, 23 and 25) with a frequency < 0.06.

**Table 1:** Details of the 22 natural populations of *Pinus cembra* in the western Alps: Situation (central vs marginal); site names of populations; ID-codes (F, France; I, Italy; geographic locations in terms of latitudes and longitudes (in decimal degrees) and mean sampling altitudes at each site (m a.s.l.); and genetic diversity indices

Situation	Population	ID	Lat.	Lon.	Alt.	n	*N*_a_	*N*_e_	*N*_p_	AR	*H*_o_	*H*_e_	*F*_IS_	HWE
Central	Bosco di Alevé (I)	CAL	44.611	7.080	2076	37	6.571	2.703	2	5.306	0.617	0.549	−0.104	0.452
	Aussois (F)	CAU	45.255	6.719	2049	29	6.429	3.318	1	5.636	0.578	0.598	0.086	0.235
	Bois des Ayes (F)	CAY	44.821	6.655	1991	31	6.571	2.770	2	5.137	0.534	0.537	0.038	0.162
	Chamonix (F)	CBL	45.917	6.897	1807	39	6.286	3.017	1	5.212	0.647	0.620	−0.044	0.133
	Lago Perso (I)	CLP	44.906	6.795	1998	30	6.286	3.126	1	5.316	0.706	0.602	−0.186	0.170
	Lanslevillard (F)	CLV	45.287	6.949	2003	28	7.000	3.302	0	5.750	0.640	0.596	−0.081	0.319
	Lac Miroir, Ceillac (F)	CMI	44.631	6.794	2279	30	6.143	2.838	0	5.047	0.667	0.581	−0.113	0.253
	Orelle (F)	COR	45.193	6.535	1722	32	6.857	3.433	0	5.645	0.713	0.614	−0.154	0.194
	La Plagne (F)	CPL	45.509	6.666	2005	30	6.429	3.360	0	5.233	0.695	0.631	−0.095	0.457
	Méribel (F)	CTU	45.364	6.587	1766	39	6.286	2.548	0	4.756	0.644	0.555	−0.133	0.272
	Serre Chevalier (F)	CSC	44.923	6.545	2140	34	6.714	3.297	0	5.816	0.697	0.655	−0.058	0.461
	Mean				1985.1	32.6	6.506	3.065	0.6	5.350	0.649	0.594	−0.077	
Marginal	Aravis-La Clusaz (F)	MAR	45.897	6.471	1884	28	5.571	2.780	0	4.628	0.673	0.558	−0.161	0.103
	Gordolasque (F)	MAU	44.075	7.403	1766	27	5.429	3.208	0	4.941	0.722	0.618	−0.156	0.477
	Vallon de la Braisse (F)	MBR	44.286	6.807	2198	24	5.714	3.267	2	5.069	0.669	0.622	−0.021	0.329
	Chamrousse (F)	MCH	45.112	5.891	1910	30	7.143	2.898	1	5.564	0.640	0.575	−0.072	0.274
	Dévoluy (F)	MDE	44.612	5.945	1791	46	7.857	3.044	3	5.998	0.520	0.574	0.115*	0.084
	Flaine (F)	MFL	46.001	6.710	2011	30	5.000	2.348	0	4.205	0.507	0.455	−0.081	0.532
	Gilly-sur-Isère (F)	MGI	45.597	6.383	1859	50	5.714	2.660	1	4.572	0.538	0.575	0.097*	0.260
	Moulières (F)	MMO	44.189	6.565	2049	29	6.571	2.648	1	5.059	0.501	0.556	0.103*	0.493
	Roya (F)	MRO	44.115	7.493	1722	29	4.286	2.441	2	3.721	0.588	0.505	−0.037	0.253
	Taillefer (F)	MTA	45.054	5.921	1946	29	6.429	2.975	0	5.319	0.610	0.571	−0.046	0.346
	Valgaudemar (F)	MVA	44.701	6.152	2073	29	6.571	3.014	0	5.624	0.685	0.628	−0.084	0.218
	Mean				1928.1	31.9	6.026	2.844	0.9	4.973	0.605	0.567	−0.031	
	Overall mean				1956.6	32.3	6.266	2.954	0.8	5.162	0.627	0.581	−0.054	
	Standard deviation				154.4	6.3	0.761	0.316	0.9	0.546	0.071	0.045	0.090	

*n, number of sampled individuals; N*_a_, number of different alleles; *N*_e_, number of effective alleles; *N*_p_, number of private alleles; AR, allelic richness; *H*_o_, observed heterozygosity; *H*_e_, expected heterozygosity; *F*_IS_, inbreeding coefficient; HWE, Hardy-Weinberg equilibrium (*p*-value), *p<0.05.

### Levels and patterns of genetic diversity

The Micro-Checker software revealed no evidence of null alleles, large allele dropout or scoring errors due to stuttering (based on 95% CI, derived from 1000 randomizations). Generally, all populations conformed to Hardy-Weinberg equilibrium (HWE) with no significant deviations and although a few loci showed some deviation, this does not appear to be associated with null alleles or non-neutral behavior. Additionally, no loci appear to be consistently linked across all populations (data not shown).

We evaluated the intrapopulation genetic diversity both in all populations and between the central and marginal groups. Allelic richness (AR), based on 95 detected alleles at seven nSSR loci, was estimated at an overall mean of 5.162; however, the mean was higher for populations in the central group (5.350) than for populations in the marginal group (4.973). The overall mean observed heterozygosity (*H*_o_) was 0.627 and expected heterozygosity (*H*_e_) 0.581. Both of these indices were slightly lower for the marginal group (0.605 and 0.567, respectively) than the central group (0.649 and 0.594, respectively). The estimated inbreeding coefficient (*F*_IS_) ranged from − 0.186 for population CLP to 0.115 for population MDE. Mean values were − 0.077 for the central group and − 0.031 for the marginal group. However, significant inbreeding was only detected in the marginal populations MDE, MGI and MMO (Table 1).

Independent sample *t*-tests did not reveal any significant difference in any of the mean genetic diversity indices (AR *t* (20) = 1.693, *p* = 0.106; *H*_o_ *t* (20) = 1.503, *p* = 0.148; *H*_e_ *t* (20) = 1.445, *p* = 0.164) and inbreeding (*F*_IS_ *t* (20) = − 1.194, *p* = 0.246) between the central and marginal groups (*N* = 11 populations in each group). In addition, Levene’s *F* test indicated that the assumption of homogeneity of variances between the central and marginal groups was met (AR *F* (20) = 3.555, *p* = 0.074; *H*_o_ *F* (20) = 2.801, *p* = 0.110; *H*_e_ *F* (20) = 0.322, *p* = 0.576; *F*_IS_ *F* (20) = 0.515, *p* = 0.481).

### Population stability

Tests of genetic bottleneck yielded conflicting results. BOTTLENECK analysis, with both the SMM and TPM models, did not reveal a signature of a historical population size reduction. Similarly, the ‘mode shift’ index test showed a normal L-shaped distribution of allele frequency for all populations, which is expected in populations that are near to mutation-drift equilibrium (Table 2). In contrast, the *M*-ratio test indicated that all populations had experienced a historical reduction in size. The population-specific *M*-ratios ranged from 0.209 (MBR) to 0.356 (MRO), and were all below the threshold of *M* < 0.680 indicating a pronounced historical bottleneck (Table 2).

**Table 2:** Results of genetic bottleneck tests for the 22 *Pinus cembra* populations

ID	SMM (*p*-value)	TPM (*p*-value)	Mode shift	*M*-ratio (*p*-value, SD)
CAL	1.000	0.988	normal L-shaped	0.221 (0.174)
CAU	0.988	0.852	normal L-shaped	0.253 (0.169)
CAY	1.000	0.988	normal L-shaped	0.215 (0.183)
CBL	0.988	0.531	normal L-shaped	0.225 (0.131)
CLP	1.000	0.813	normal L-shaped	0.252 (0.183)
CLV	1.000	0.988	normal L-shaped	0.217 (0.170)
CMI	0.996	0.988	normal L-shaped	0.242 (0.174)
COR	0.992	0.852	normal L-shaped	0.275 (0.151)
CPL	0.961	0.344	normal L-shaped	0.239 (0.172)
CTU	1.000	1.000	normal L-shaped	0.257 (0.168)
CSC	0.988	0.766	normal L-shaped	0.262 (0.151)
MAR	0.992	0.945	normal L-shaped	0.256 (0.191)
MAU	0.711	0.344	normal L-shaped	0.293 (0.157)
MBR	0.766	0.766	normal L-shaped	0.209 (0.175)
MCH	1.000	0.996	normal L-shaped	0.263 (0.172)
MDE	1.000	0.996	normal L-shaped	0.246 (0.165)
MFL	1.000	0.988	normal L-shaped	0.228 (0.176)
MGI	0.996	0.766	normal L-shaped	0.227 (0.178)
MMO	1.000	0.988	normal L-shaped	0.235 (0.166)
MRO	0.945	0.469	normal L-shaped	0.356 (0.190)
MTA	1.000	0.961	normal L-shaped	0.248 (0.157)
MVA	1.000	0.988	normal L-shaped	0.266 (0.155)

SMM, Stepwise Mutation Model; TPM, Two Phase mutation Model

*p*-value, probability according to one-tailed Wilcoxon signed-rank test of heterozygote excess

### Genetic differentiation and structure

**Fig. 2:**
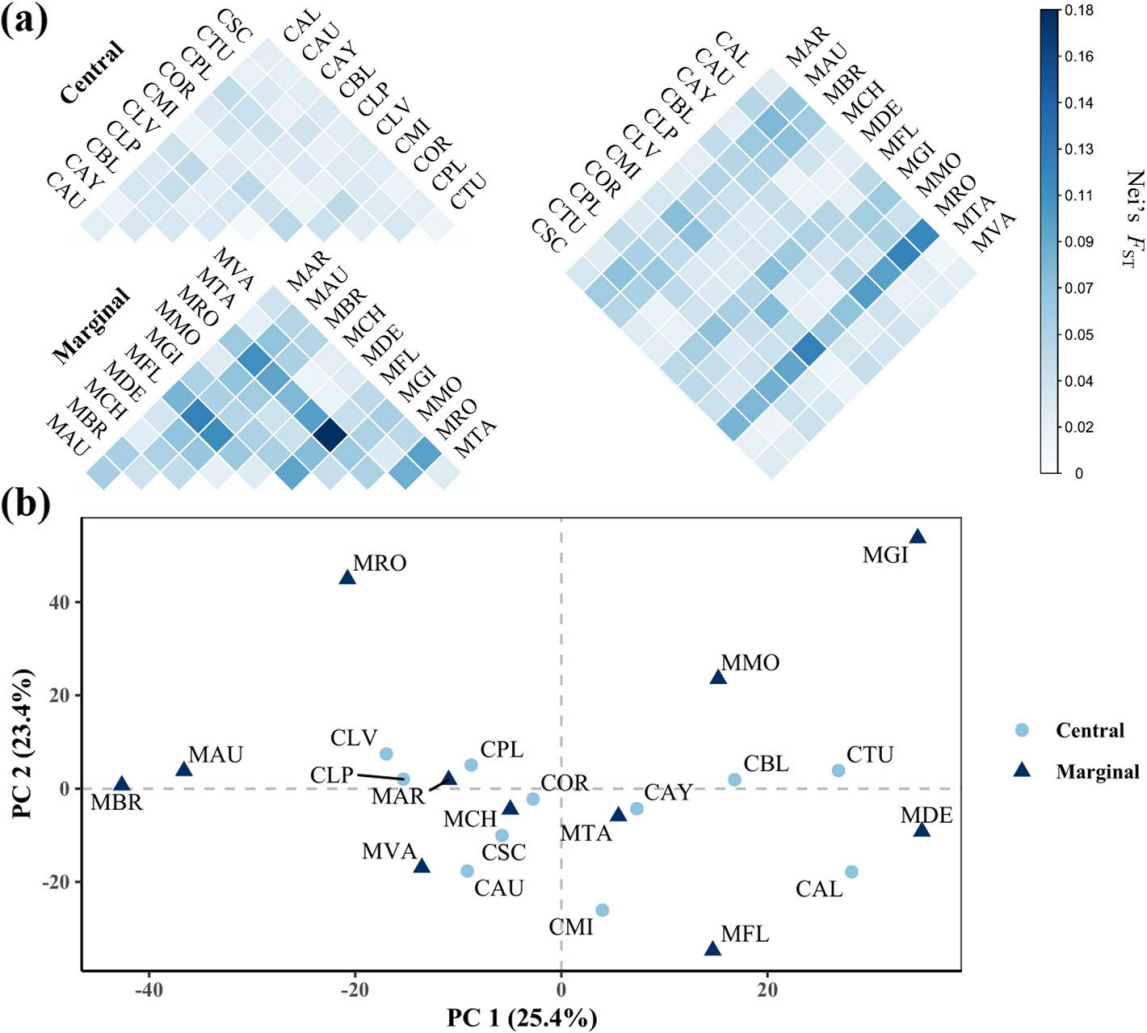
Patterns of genetic differentiation: **a** Matrix of pairwise *F*_ST_ values between the 22 cembra pine (*Pinus cembra* L.) populations. Colors representing Nei’s genetic distances are defined on the scale at the right side of the figure. **b** Two-dimensional plot of the two main principal components (PC) and their part of the total variance in % using Principle Component Analysis (PCA). Population abbreviations are as explained in Table 1

Significant genetic differentiation was detected between population pairs and between geographic regions. AMOVA of data for all populations yielded an *F*_ST_ value of 0.065 (*p* < 0.01), indicating that differentiation among populations accounts for > 6% of the total genetic variance. However, the regional comparison indicated that differentiation between central and marginal populations accounts for just 1% of the total variation (*F*_ST_ = 0.009 *p* < 0.01; Table 3). All the pairwise between-population *F*_ST_ values were > 0 (0.010 to 0.180), indicating the presence of population structure in *P. cembra*. In addition, the pairwise *F*_ST_ values were substantially higher for marginal populations (mean *F*_ST_ 0.096 ± 0.115) than for central populations (mean *F*_ST_ 0.027 ± 0.027) (Fig. 2a, Additional file 1: Table S3). Similarly, PCA indicated discernible genetic differentiation in majority of marginal populations, notably MBR, MAU, MRO, MFL and MGI, and only few of them (MAR, MCH, MTA and MVA) intermingled with the central group (Fig. 2b). The estimated overall average gene flow parameter (Nm) for all populations was 3.648, and the value was higher for central populations (6.622) than for marginal populations (2.614).

**Table 3:** Results of analysis of molecular variance (AMOVA) between regions and populations of *Pinus cembra*.

Spatial scale	Source of variation	D.f.	Sum of square	Mean square	Estimated variance	Variance (%)	Fixation index (*F*_ST_)
Pop vs. Pop	among pop.	21	256.352	12.207	0.152	6%	0.065**
	within pop.	1432	3130.567	2.186	2.186	94%	
	total	1453	3386.920		2.338	100%	
Central vs. Marginal	among regions	1	16.642	16.642	0.020	0.8%	0.008**
	within regions	1452	3370.277	2.321	2.321	99.2%	
	total	1453	3386.920		2.341	100%	

significance calculated with 999 permutations: **p < 0.01

**Fig. 3:**
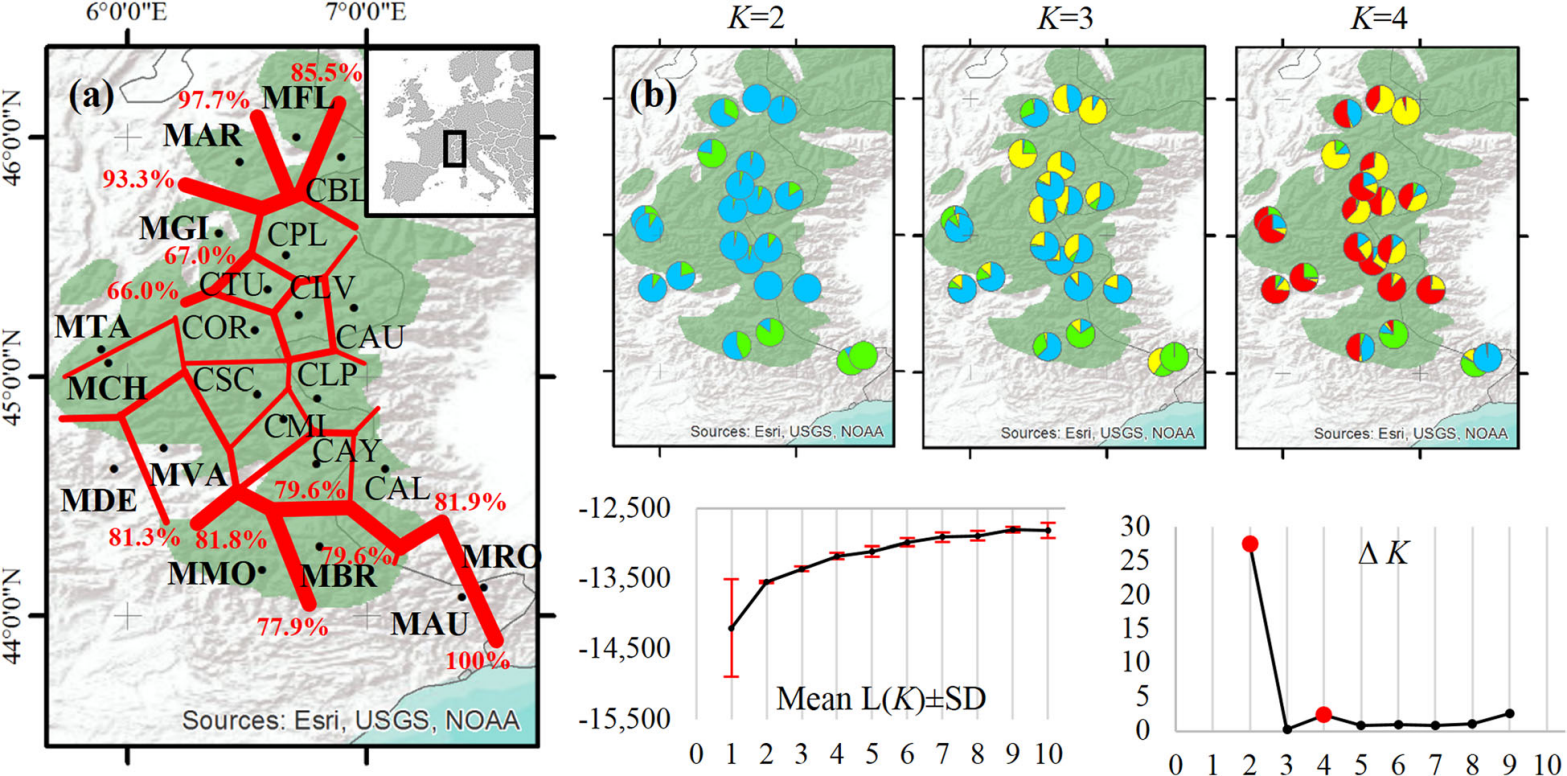
Results of Barrier analysis of genetic discontinuities among, and structure of, the 22 cembra pine (*Pinus cembra*) populations: **a** Genetic delimitations in the spatial distribution of populations, visualized with red lines with indicated bootstrap support (%). **b** Estimated population structures for *K* = 2, *K* = 3 and *K* = 4 genetic groups, based on Mean L(*K*) (±SD) and Δ*K* values. Population abbreviations are as explained in Table 1. The natural distribution of cembra pine according to the EUFORGEN (2018) database is marked in dark green

Structure analysis was applied to determine the optimal numbers of genetic groups and subgroups for explaining the variation in nSSR data. The highest Δ*K* value was obtained when *K* = 2, and there was a second, albeit lower, Δ*K* peak when *K* = 4, indicative of population substructuring (Fig. 3). The plotted cluster memberships (Q-matrix) at each *K* value from *K* = 2 to *K* = 4 revealed relatively high levels of genetic admixture and no sharp segregation within the western Alps. This is particularly clear for the central group when *K* = 4. However, marginal populations are largely differentiated and generally belong to separate and distinct groups (Fig. 3). Barrier analysis provided similar results as genetic discontinuities with 66 to 100% bootstrap support were only detected between marginal populations. These populations were situated away from the central group at the northwestern and southern range margins. All putative barriers between the central populations were weak, with < 43% bootstrap support, indicating non-significant separation (Fig. 3).

### Isolation by distance and climatic heterogeneity

**Fig. 4:**
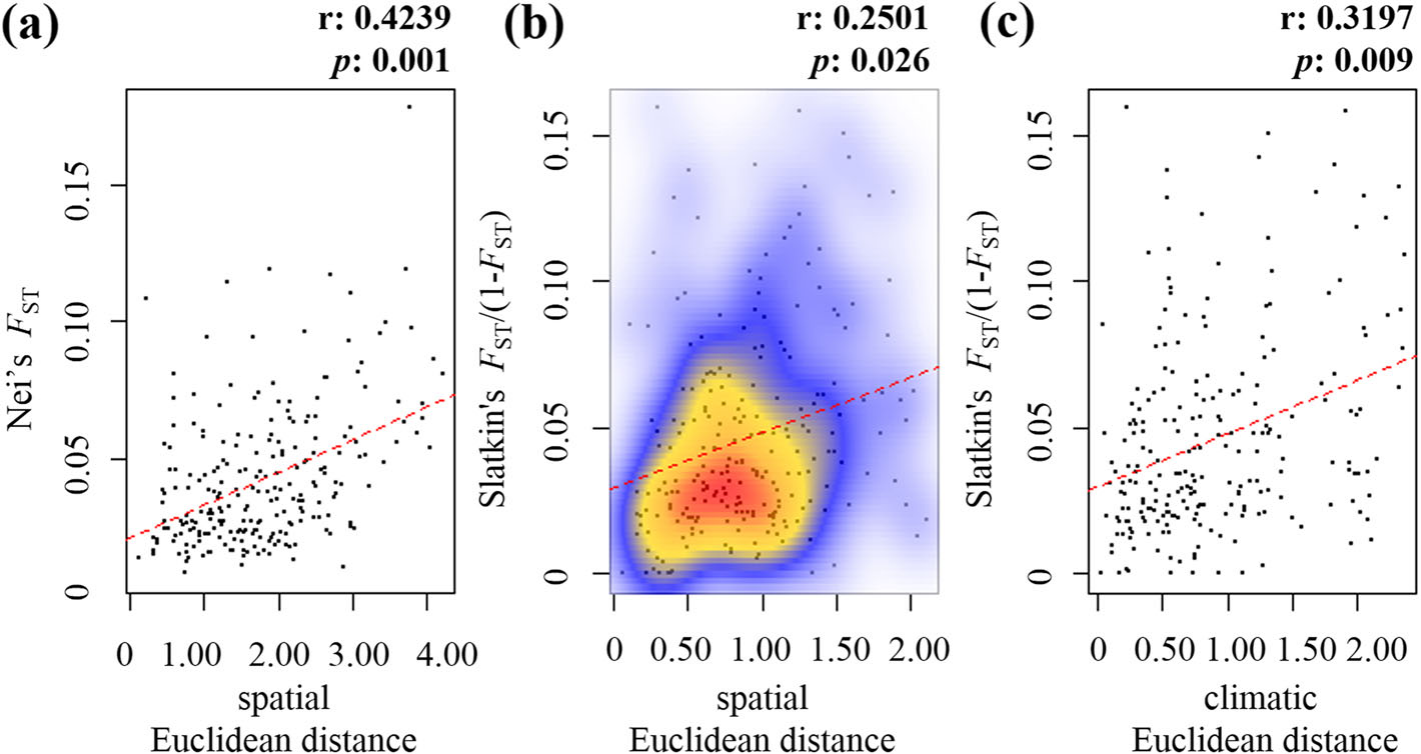
Results of Mantel tests of correlations: **a** between genetic differentiation (Nei’s *F*_ST_) and geographic distance (spatial Euclidean), **b** between genetic differentiation (Slatkin’s linearized *F*_ST_) and geographic distance (spatial Euclidean) with 2-D kernel density estimation, **c** between genetic differentiation (Slatkin’s linearized *F*_ST_) and climatic distance (climatic Euclidean)

We found that both geographic and climatic distances contribute significantly to genetic differentiation between populations of cembra pine in our study area (Table 4, Fig. 4). A Mantel test including data for all sampled individuals indicated significant differentiation in line with increasing geographic distance: Nei’s *F*_ST_ *R* = 0.42, *p* = 0.001 (Slatkin’s linearized *F*_ST_: *R* = 0.25, *p* = 0.026). In addition, scatterplots showing local densities of distances (Fig. 4) revealed a continuous cline of genetic differentiation with no sign of discontinuity. A Mantel test between genetic and climatic distances revealed these variables were significantly correlated (*R* = 0.32, *p* = 0.009). Similarly, partial-Mantel tests using *F*_ST_ as the constant variable indicated a significant correlation with geographic distance when controlling for climatic distance (*R* = 0.27, *p* = 0.011), and with climatic distance when controlling for geographic distance (*R* = 0.31, *p* = 0.020). However, the regression coefficient was larger for climatic distance than for geographical distance (*R* = 0.31 and *R* = 0.27, respectively). MMRR analysis resulted in a highly significant correlation between *F*_ST_ and the climatic distance matrix (*R* = 0.34, *p* = 0.002), while the contribution of geographic distance to genetic differentiation was non-significant, only if evaluated with altitudinal distance (Table 3).

**Table 4:** Results of standard Mantel tests, partial-Mantel tests and Multiple Matrix Regression with Randomization (MMRR) analyses

Test	Parameters	R	*β*	*p*
Mantel	Gen vs. Geo	0.423	–	0.001***
	Gen vs. Clim	0.319	–	0.009***
partial-Mantel	Gen vs. Geo (Clim)	0.269	–	0.011**
	Gen vs. Clim (Geo)	0.313	–	0.020**
MMRR	Gen vs. Geo + Clim	0.342	Geo: 0.002	0.531^ns^
			Clim: 0.004	0.002***
	Gen vs. Geo + Alt	0.200	Geo: 0.011	0.001***
			Alt: 0.004	0.205^ns^

significance calculated with 999 permutations: **p < 0.05, ***p < 0.01, ns; not significant

Gen, genetic distance (F_ST_); Geo, geographic distance; Clim, climatic distance partial-Mantel tests: X ~ Y(Z) is the correlation between X and Y matrices, controlling for Z

## Discussion

Our results suggest that, regardless of their location within the western Alps, cembra pine populations maintain substantial amounts of genetic variation at near to mutation-drift equilibrium, with low intrapopulation inbreeding. However, marginal populations differ from those at the center of the range and have both higher genetic differentiation and restricted gene flow. In addition to geographic and genetic isolation, climatic heterogeneity is a key driver of differentiation among marginal populations. To increase our understanding of these processes, we quantified the genetic diversity and differentiation among populations in the central and marginal ranges and explored discontinuities in the genetic pattern across the surveyed area. Finally, we used multiple matrix correlations and regression analyses to infer effects of recent environmental heterogeneity on genetic differentiation among the populations.

### Marginal populations have maintained their genetic diversity

Generally, genetic diversity is expected to decrease towards margins of species’ ranges due to factors including intensified fragmentation, isolation, restricted gene flow and genetic drift [36, 37]. Similarly, the ‘central-marginal’ theory predicts that marginal populations are less genetically diverse because they are generally smaller, more sparsely distributed, and experience less favorable ecological conditions [3, 38, 39]. However, our results did not support this hypothesis of lower genetic diversity in marginal populations. Although genetic diversity (measured by AR, *H*_o_ and *H*_e_) was slightly lower in marginal populations than in central populations, these differences were non-significant. For example, mean expected heterozygosity (i.e. gene diversity) was 0.567 in marginal and 0.594 in central populations, which is not significantly different (*p* < 0.05). Furthermore, the overall mean *H*_e_ for the 22 populations was 0.581, which is slightly higher than values previously obtained for *P. cembra* populations using the same set of nSSR markers: 0.505 in the eastern Alps, and 0.547 [40] and 0.564 [29] in the Carpathians. This high genetic diversity indicates that regardless of their location in the western Alps, *P. cembra* populations appear to have preserved genetic variation and avoided large scale genetic erosion.

Several factors could promote high genetic diversity, including several common features of many conifers, such as longevity, an outcrossing mating system, wind-dispersed pollen and high fecundity [41]. In addition, range geometry, orientation, latitude, ecological factors, phylogeographic history and postglacial range dynamics can influence a species’ modern-day genetic diversity [42–44]. Although factors such as these have probably affected cembra pine populations in the western Alps, they have not decreased their overall genetic diversity.

We did not detect any substantial deviations from Hardy-Weinberg equilibrium (HWE) and intrapopulation inbreeding (*F*_IS_) values were slightly negative or around zero, indicating no heterozygote deficiency and thus no inbreeding. Additionally, mean *F*_IS_ values did not differ significantly between the C and M regions. Interestingly, inbreeding depression was previously reported in Carpathian populations [29, 40], suggesting that restricted connectivity to surrounding populations, enhanced by factors such as limited pollen and seed dispersal, had resulted in selfing and mating between relatives. However, these studies exclusively investigated isolated and sporadic populations, whereas marginal populations located in the western Alps are geographically closer and more linked to populations in the central part of the range, which reduces inbreeding. Significant inbreeding coefficients were only obtained for three populations in the marginal group (MDE, MGI and MMO). This might be a consequence of the spatial segregation and isolation that is commonly observed and expected in small, fragmented and marginal populations [11]. Alternatively, since the MGI, MDE and MMO populations are very small and located in an isolated landscape, it might be the consequence of local landscape features (e.g., topography) restricting regional gene flow.

### Historical bottleneck fits the paleoecology, but recent population sizes are stable

We obtained conflicting results from the two population stability tests (bottleneck and *M*-ratio), applied to detect historical reductions (if any) in population size. First, bottleneck analysis using both SMM and TPM models detected no evidence of a significant excess of heterozygosity in any population. Moreover, the mode-shift indicator detected a normal L-shaped allele frequency distribution, as expected for populations that are near to mutation-drift equilibrium. Based on this allele frequency distribution, we can assume that present day populations are randomly mating. However, the Garza-Williamson indices (*M*-ratios; all < 0.43) suggested that all present-day populations are remnants of a substantially larger population [45, 46]. Combinations of < 0.68 *M*-ratios and lack of heterozygosity excess are regarded as indicative of severe, old bottlenecks, while significant heterozygosity excess is expected following weak, relatively recent (within a few millennia) bottlenecks [47, 48]. Thus, our results indicate that populations in the western Alps may have experienced a severe historical bottleneck, but more recently population sizes have remained relatively stable. Paleoecological evidence suggests that subalpine forests in the western Alps began to decline in size around ca. 6000 cal yr BP [49–51], and by 2500 cal yr BP had significantly contracted. These trends have been associated with increases in human activities in the high Alpine mountains, particularly increases in clearance of forests by fire for domestic herd grazing [52–54], promoting subalpine grasslands or *Larix*-type forests [55, 56]. This degradation of cembra pine communities, which began in the mid-Holocene and dramatically reduced the overall population size, may have resulted in a severe historical bottleneck. More recently, population sizes seem to have remained relatively stable, as we found normal levels of heterozygosity and allele frequency distributions. In contrast to the *M*-ratio, which is expected to have a long recovery time, heterozygosity excess and allele frequency distributions will recover relatively quickly when conditions allow the addition of new and rare alleles [57]. If populations are randomly mating at HWE with no signals of inbreeding, as we observed, we can conclude that populations are currently stable.

### Genetic differentiation and population structuring towards the range margin

Both the structure analysis and pairwise *F*_ST_ values indicated that genetic structure and differentiation significantly increase towards the margin of the species’ range. Central populations are highly admixed and homogenous with no significant differentiation, while populations at the margin are genetically more isolated and heterogeneous. Barrier analysis supported these results and revealed no genetic discontinuities among central populations, but several barriers (with high bootstrap support) between the marginal populations, especially at the northwestern and southern limits of the range. This pattern was corroborated by higher values of the gene flow parameter Nm for the marginal group than for the central group.

Our findings are consistent with the ‘central-marginal’ theory, which predicts a general increase in genetic differentiation towards margins of species’ ranges [1]. High levels of genetic differentiation can also explain the high levels of genetic diversity we observed, because disproportionately high levels of differentiation between nearby populations can lead to exceptionally high levels of regional genetic diversity [3, 58, 59]. We assume that multiple factors have contributed to the present-day genetic structure, particularly those associated with past demography and environmental variability.

### Signatures of glacial refugia and postglacial migration routes

Paleoecological evidence suggests that *P. cembra* most likely survived the Last Glacial Maximum (LGM) near or within the western Alps [30, 31, 35, 60], notably near to the site of our CMI population. This suggests that species could have also survived at multiple other locations in the southern Alps during glacial periods [27]. This scenario was previously postulated on the basis of genetic [25] and paleoecological evidence [26, 60]. Our structure analysis indicated the presence of at least two groups and most likely four subgroups of *P. cembra* populations (Fig. 3b). In analyses assuming two, three or four subgroups, the four southernmost populations (MBR, MMO, MAU, MRO) were distinguished from the other 18. In particular, when *K* = 2 the remaining 18 populations, with the exception of northern populations near the Swiss boundary, appear to be generally less differentiated and more homogeneous. When *K* = 3 or 4, these 18 populations appear as an admixture of two provenances that could support one or both of the following migrations routes: one from Switzerland (the “Classic” scenario), and one from the east though the Italian plains or foothills (the “Southeastern Alpine” scenario, [25]. The four southernmost populations could have origins in previously postulated “Intra-Alpine southern refugia” [27]. Interestingly, a mitochondrial-based genetic study on *Larix decidua* in Europe revealed a marginal haplotype (H22) that was dominant in one population of the southern Alps [61] at a site exactly corresponding to the location of our southern marginal population MBR, which was clearly separated in the structure analysis. This convergence of genetic pattern at the southern tip of the Alps supports the existence of common glacial and postglacial refugia for larch and cembra pine in these southern Alps or surrounding areas in France or Italy. Accordingly, these southern Alps were only partly covered by glaciers and many *nunataks* emerged [62, 63], which could have harbored subalpine-type communities in glacial refugia [27] and subsequently populations could have recolonized the Alpine foothills during the Lateglacial period that lasted from 18,000 to 11,700 cal yr BP.

Regardless of the postglacial migration route, it is therefore likely that present-day marginal populations were connected to central populations during the postglacial expansion of *P. cembra* (before 9000 cal yr BP). Micro- and macro-remains indicate that the species’ abundance in the subalpine belt peaked between 9000 and 6000 BP, and in the mid-Holocene *P. cembra* became a common tree at high elevations in both the marginal and central western Alps, as shown (for example), by [51, 64], respectively. The previously widespread distribution of *P. cembra* started to decline between 6000 and 3000 cal yr BP, depending on the favorability of its mountain habitats for human societies, in terms (for example) in distance from existing settlements, slopes, exposure or rock cover. These human-driven processes are likely to have promoted further fragmentation, segregation and decline in population sizes, especially at the margins of the species’ range. Indeed, the first postglacial fragmentation processes began towards the end of the Lateglacial period and the early Holocene (11,700–8000 cal yr BP), when a warming climate drove migration of cembra pine from peripheral plains and central valleys to higher altitudes, thus disconnecting marginal and central populations. Peripheral fragmented populations are often prone to severe stress because of the temporal and spatial variation of the environment, demographic stochasticity and edge effects, such as genetic isolation and drift [13, 14]. Thus, marginal *P. cembra* populations could have separately evolved genetic variation, ultimately resulting in the observed genetic differentiation.

Alternatively, landscape heterogeneity and environmental variation can generate fluctuating or contrasting selection regimes that affect patterns of gene flow and ultimately spatial population genetic structure [65, 66]. In addition, marginal populations generally grow in less optimal environments than central populations, and may evolve adaptations to specific climatic or edaphic stresses [67]. Nevertheless, marginal populations, for example those near the northern forelands of the French Alps (where the climate is relatively wet and oceanic), or the southern tip of the western Alps (where the climate is more Mediterranean), may be particularly sensitive to regional climate fluctuations. For example, marginal populations in the south may be highly prone to drought stress and temperature rises, because they are already close to their drought and temperature limits, while northwestern populations may be highly sensitive to increases in precipitation. Climate-growth models have already revealed that conifer species of mountain environments (*Abies alba*, *Pinus sylvestris* and *Pinus uncinata*) and boreal regions (*Pinus koraeiensis* and *Thuja occidentalis*) located at margins of their respective ranges are particularly vulnerable to climatic changes (e.g. [68–70]. This is also expected for *P. cembra*, where populations in the southern, Mediterranean climatic region reportedly have high sensitivity to precipitation [71], and the climate is predicted to become increasingly arid. Climate change in this region is expected to exacerbate soil moisture and vapor pressure deficits in forest ecosystems [72, 73]. Accordingly, environmental variability could drive local selection processes acting on standing neutral variation and could create or increase genetic differentiation between marginal populations, while in central populations land use abandonment since the nineteenth century favors *P. cembra* recruitment [74, 75] and may help to reduce demographic effects on genetic differentiation.

### Climatic heterogeneity is a stronger driver of isolation than geographical distance

Mantel and partial-Mantel tests detected significant correlation between *F*_ST_ and geographical distances between the populations, indicating that genetic differentiation among the populations significantly increases with geographic distance. The ‘isolation by distance’ (IBD) theory states that if dispersal (i.e. gene flow) between regions or populations is geographically restricted, genetic differences can accumulate locally [76, 77]. Our results largely confirm this theoretical consideration, since we identified restricted gene flow, barriers and extensive population structuring, particularly in marginal *P. cembra* populations. In addition, while marginal populations in the western Alps are spaced out along the edge of the distribution, central populations are close to each other, thereby promoting higher gene flow between the stands, which further supports IBD. Although previous studies found only weak or non-significant IDB [25, 28, 29], their estimations of regional gene flow follow this pattern. It is likely that populations in the central Swiss Alps are larger and more connected, and have experienced less genetic drift and more frequent pollen or seed dispersal, whereas the Carpathian populations are highly fragmented and spatially isolated, which severely limits gene flow [29, 40].

However, partial-Mantel tests and MMRR analysis suggest that climatic constraints have an even stronger influence on genetic differentiation than geographical distance, in accordance with ‘isolation by environment’ (IBE) theory, i.e., that genetic differentiation increases with environmental differences [78–80]. Our partial-Mantel tests indicate that *F*_ST_ is more strongly correlated with climatic distance than with geographical distance (*R* = 0.31 and *R* = 0.27, respectively). Furthermore, MMRR analysis indicated a significant linear relationship between climatic distances. Together our partial-Mantel and MMRR results suggest that IBE explains most of the genetic differentiation, although IBD also contributed significantly. Alternatively, meaning that genetic divergence resides among far spaced populations inhabiting different environments with contrasting ecological conditions [19, 21, 81].

Our results suggest that *P. cembra* populations inhabiting the western Alps, especially the marginal regions, are highly affected by regional topographical features of the Alps (such as isolated mountain massifs) that control gene flow, and microclimatic conditions that influence population genetic processes such as selection that can contribute to population divergence. Previously, the climate-topography relationship has been widely studied in the Alps and the results have highlighted the substantial effect of climate variability [82, 83]. Moreover, the influence of climate on genetic variation has been investigated for various alpine plant species, including *P. cembra,* and the results indicate that precipitation and temperature predominantly influence genetic differentiation and structure [84–86]. Together, these results suggest that local environmental factors are likely to act as major selective drivers of local genetic differentiation and in addition to past historical events may underlie the current population structure of *P. cembra* in the western Alps.

## Conclusions

Our study suggests that both central populations, situated along the main axis of the western Alps, and marginal populations at the edge of the species’ range, can maintain stable sizes over long periods and preserve high amounts of genetic variation. Surprisingly, there was no significant partitioning of genetic variation within the investigated area and isolated populations did not develop substantial inbreeding, indicating that there is recurrent gene flow between modern-day populations through seed and/or pollen distribution. However, there is clear regional-scale genetic differentiation and structuring of *P. cembra*. While central populations are homogenous with high admixture, the fragmented marginal stands are structured, heterogeneous and geographically-genetically isolated. The highest degree of genetic differentiation is present in populations at the margins of the species’ range, partly due to their geographical distance from central populations, but mainly due to climatic heterogeneity. In addition to the demographic history, environmental differences between the central and marginal regions, and between populations, may also have contributed to current levels of genetic diversity and divergence in *P. cembra*. Finally, our results support a published hypothesis that *P. cembra* survived in multiple refugia within the western Alps.

## Methods

### Population sampling and regional setting

Twenty-two natural cembra pine populations, consisting 11 central (C) and 11 marginal (M) populations, were selected from French and Italian Alps and classified based on their geographical position, isolation, size and habitat characteristics (Table 1). The central populations are located well within the range of the species along the main inner French-Italian axis of the Alps and are major components of large forests that are close to each other, while marginal populations generally cover smaller, highly fragmented areas at low densities, isolated from central populations. One-year-old needles were sampled from 727 mature trees (24–46 from each natural population), at a mean altitude of 1956 ± 154 m a.s.l. (Table 1), spaced at least 30 m apart to avoid sampling closely related individuals. Needles were stored on silica gel and frozen at − 80 °C until DNA extraction.

### Microsatellite genotyping

Total genomic DNA was extracted from 20 to 25 mg samples of plant material using a DNeasy 96 Plant Kit (Qiagen, Valencia, CA, USA) following the manufacturer’s instructions. Seven polymorphic microsatellite (nSSR) loci (pc1b, pc3, pc7, pc18, pc22, pc23, and pc25) developed by [87] were used to genotype all the trees collected at the sampling locations. Forward nuclear primers were fluorescently labelled with 6-FAM (pc1b, Pc3, Pc7), NED (Pc18), PET (Pc22, Pc25) and VIC (Pc23). The microsatellite loci were amplified by PCR [87], and the products were checked by electrophoresis in a 1% (w/v) GelRed-stained (Biotium, Hayward, CA, USA) agarose gel in 1× TBE buffer. Finally, PCR fragments were sized by Fragment Length Analysis (FLA) using an Automated Capillary DNA Sequencer (ABI 3130, Applied Biosystems, Foster City, CA, USA). Genotypes were determined using GeneMapper Software v.4.1 (Life Technologies, Carlsbad, CA, USA). Each genotype was visually checked, scored and unclear samples were reamplified and rescored. All loci were checked for the occurrence of null alleles, large allele dropout, and stutter bands with Micro-Checker software [88].

### Statistical data analysis

#### Genetic diversity

Genetic diversity indices, including the number of different alleles (*N*a) and number of effective alleles (*N*e), were calculated for each population using GenAlEx v.6.5 software [89]. The number of private alleles (*N*p) and allelic richness (AR) were computed in R [90] using the “popgenreport” package [91]. The expected heterozygosity (*H*_e_), observed heterozygosity (*H*_o_), and inbreeding coefficient (*F*_IS_) were calculated using the “poppr” package [92], while the significance of *F*_IS_ values was calculated separately with FSTAT software [93] using 1000 permutations. Deviation from Hardy-Weinberg equilibrium (HWE) was tested locus-by-locus for each population by *F*_IS_ statistics [94] using GENEPOP version 4.7.0 [95]. For this analysis we set the Markov chain parameters to dememorization = 10,000, batches = 20, and iterations per batch = 5000. To determine whether the parameters of genetic diversity (AR, *H*_o_, *H*_e_) and inbreeding (*F*_IS_) differed significantly between the central and marginal groups of populations we applied independent sample two-tailed *t*-tests in SPSS v.22 (SPSS Inc., Chicago, IL, USA). To test whether distributions of variables met normality requirements, we applied one-sample Kolmogorov-Smirnov tests, and where necessary variables were log-transformed. Throughout the analysis, 95% confidence intervals (CI) and bootstrapping with 1000 replicates were applied. The homogeneity of variances assumption was tested with Levene’s *F* test for all variables.

### Population stability analysis

We used two approaches to investigate population stability. First, we tested for evidence of population bottlenecks by calculating the heterozygote excess relative to the number of alleles using BOTTLENECK 1.2.02 [96], which correlates the expected heterozygosity (*H*_e_) and observed heterozygosity (*H*_o_) at mutation-drift equilibrium. For this, as well as the most conservative Stepwise Mutation Model (SMM), we also applied the Two-Phase Model (TPM) allowing multiple-step mutations, which is recommended for microsatellite data [97, 98]. The TPM model assumes a distribution of 30% of multiple-step mutations and 70% single-step mutations. For each population, 2000 simulations were performed and the significance was assessed using the implemented Wilcoxon sign-rank test. In addition, the ‘mode shift’ qualitative descriptor of allele frequency distribution was applied to discriminate between bottlenecked and stable populations [99]. In a second test, we calculated the Garza-Williamson index (*M* or *M*-ratio), which is the number of alleles (k), divided by the allelic range (r), using Arlequin v.3.5 [100]. The calculated value is expected to decrease proportionally in line with the severity and duration of a population size reduction [45], and generally *M* < 0.680 is regarded as indicative of a pronounced historical bottleneck [45, 101].

### Genetic differentiation and population structure

We used hierarchical analysis of molecular variance (AMOVA), implemented in Arlequin software, to determine the partitioning of the genetic variation among genetic groups, within populations, and among populations. We also compared central and marginal population groups using regional-level AMOVA. The significance of differences was evaluated using a permutation approach with 999 replications. In addition, a pairwise *F*_ST_ matrix [102] and a Principle Component Analysis (PCA) was applied using the “hierfstat” [103] and “FactoMineR” [104] packages in R to compare genetic differentiation among populations. We calculated the average gene flow estimator (Nm), which is the number of migrants per generation based on *F*_*ST*_ values, between all populations (globally), and between populations in the central and marginal groups using GenAlEx software.

To investigate the spatial genetic structure, and identify groups or subpopulations within the nSSR dataset, we implemented a Bayesian clustering approach using STRUCTURE 2.3.4 [105]. The analysis was performed using an admixture model with correlated allele frequencies. We set the *K* value (the estimated number of genetic groups) to 1–10 with a burn-in period of 10^5^ iterations followed by 10^6^ Markov Chain Monte Carlo (MCMC) steps, with 20 repetitions for each run. Subsequently we used the Evanno method [106] implemented in STRUCTURE HARVESTER Web v.0.6.94 [107] to detect the *K* value that best explained the data. Finally, the average matrices of individual membership proportions for each population were estimated using CLUMP v.1.1.2 [108] and the cluster membership coefficient of each population (Q-matrix) was plotted on a topographic map using ERSI ArcGIS (ArcMap 10.2.2, Redlands, CA, USA).

To identify sharp genetic discontinuities and spatial segregation between populations we applied Monmonier’s maximum difference algorithm, implemented in Barrier 2.2 [109]. We produced 1000 *D*_A_ distance matrices (Nei’s chord distance [110];) in Microsatellite Analyzer (MSA) [111] by bootstrapping over the seven loci. These matrices were subsequently used to estimate possible species boundaries as follows. First, the algorithm connects the spatial geographic coordinates with Delaunay triangulation and projects the corresponding Voronoi tessellations. Subsequently it traces a barrier (i.e. a predicted species boundary) along the Voronoi tessellations and assesses the robustness of the identified boundaries.

### Climatic heterogeneity

We used three different strategies to evaluate the effect of geographical isolation (IBD; isolation-by-distance) and present environmental conditions on the pattern of genetic differentiation (IBE; isolation-by-environment) of *P. cembra*. First, since marginal populations positioned separately, considerably far apart from the central population group we tested the correlation between geographical distances (kilometers) and genetic distances (Nei’s) between population pairs, IBD [76]. We generated dissimilarity matrices and evaluated them with a Mantel test [112] implemented in the “adegenet “package in R [113]. In addition, since IBD can result in either continuous clines of genetic differentiation, or in existence of distant and differentiated patches, we applied a 2-dimensional (2-D) kernel density estimator to the linearized *F*_ST_ values using the “MASS” package [114]. The kernel density approach looks for an underlying genetic structure that may help to explain observed correlation between the two distances. The *P*-value was calculated using a Monte Carlo simulation with 999 permutations. Second, to evaluate the relationships between pairwise *F*_ST_ and geographic/environmental distances we conducted a partial-Mantel test while controlling for environmental/geographic distances using the “vegan” package in R [115]. The Euclidean environmental distances were calculated from recent (c. 1950–2000) climate data using 19 bioclimatic variables (Additional file 1: Table S4), which were extracted from the global climate layer data using a grid size of 30 arc-seconds and downloaded from the WorldClim v.1.4 database (http://www.worldclim.org/). Finally, we applied Multiple Matrix Regression with Randomization analysis (MMRR [81]; the R script is deposited in the Dryad Data Repository under 10.5061/dryad.kt71r), to investigate the relative contributions of geographic and environmental (hereafter: climatic) distances to genetic differentiation (the IBE hypothesis) [80]. To perform the MMRR, we subjected the climatic data to Principal Component Analysis (PCA) using the “FactoMineR” and “factoextra” packages in R [104, 116]. Population scores of the first two vectors, which explain 86.8% of the variation for present climatic conditions, were extracted and used in the MMRR (Additional file 1: Table S5). Contribution of bioclimatic variables to each axis of PCA is reported in Additional file 1: Figure S1. Before analysis, geographical and climatic distances were standardized by zero-mean normalization.

## Supplementary information

**Additional file 1:** Supplemental materials.

## Data Availability

The climatic data are available through the WorldClim Global Climate Database from University of California, Berkeley. http://www.worldclim.org/ Microsatellite genotypes have been submitted to the TreeGenes database (08.13.2019, Accession no. TGDR158).

## Abbreviations

AMOVA: Analysis of molecular variance
AR: Allelic richness
cal yr BP: Calibrated years before present (present = 1950 AD)
FIS: Inbreeding coefficient
FLA: Fragment length analysis
FST: Fixation index (genetic differentiation)
He: Expected heterozygosity
Ho: Observed heterozygosity
HWE: Hardy-Weinberg equilibrium
IBD: Isolation-by-distance
IBE: Isolation-by-environment
K: Cluster membership probability
LGM: Last Glacial Maximum
MCMC: Markov Chain Monte Carlo
MMRR: Multiple Matrix Regression with Randomization
Nm: Number of migrants (gene flow parameter)
nSSR: Nuclear microsatellite
SMM: Stepwise mutation model (mutation)
TPM: Two-phase model (mutation)
