# Supplementary material for: Geographic isolation and climatic variability contribute to genetic differentiation in fragmented populations of the long-lived subalpine conifer Pinus cembra L. in the western Alps
Source: BMC Evol Biol. 2019 Oct 17;19:190. doi: 10.1186/s12862-019-1510-4 (PMC6798344; doi:10.1186/s12862-019-1510-4)
Supplement: Supplementary file 1 — Additional file 1: Supplemental materials. [file 12862_2019_1510_MOESM1_ESM.docx]

**Additional file 1**

Supplementary information

**Geographic isolation and climatic variability contribute to genetic differentiation in fragmented populations of the long-lived subalpine conifer *Pinus cembra* L. in the western Alps**

Endre Gy. Toth^1,2,*^, Francine Tremblay^1^, Johann M. Housset^3,4,5^, Yves Bergeron^1,3^, Christopher Carcaillet^4,6^

^1^University of Quebec in Abitibi-Témiscamingue (UQAT), Forest Research Institute (IRF),

445 Boul. de l'Université, Rouyn-Noranda, QC J9X 5E4, Canada, [yves.bergeron@uqat.ca](mailto:yves.bergeron@uqat.ca), [francine.tremblay@uqat.ca](mailto:francine.tremblay@uqat.ca)

^2^National Agricultural Research and Innovation Center (NARIC), Forest Research Institute (FRI), 9600, Várkerület u. 30/A, Sárvár, Hungary, [toth.endre@erti.naik.hu](mailto:toth.endre@erti.naik.hu), [endretoth@hotmail.com](mailto:endretoth@hotmail.com)

^3^University of Québec in Montréal (UQAM), Centre for Forest Research (CEF), C.P. 8888, succ. Centre-ville, Montréal, QC H3C 3P8, Canada

^4^Paris Sciences & Lettres University (PSL), Ecole Pratique des Hautes Etudes (EPHE), Paris, France, [christopher.carcaillet@ephe.psl.eu](mailto:christopher.carcaillet@ephe.psl.eu)

^5^Alcina, 10 rue des Amaryllis, 34070, Montpellier, France,

[johann.housset@alcina.fr](mailto:johann.housset@alcina.fr)

^6^Laboratory for Ecology of Natural and Anthropised Hydrosystems (UMR 5023 CNRS), Université Lyon 1, Villeurbanne Cedex, France

* Corresponding author

**Dates of first occurrences of *Pinus cembra* in the Alps and northern Italy before 9000 cal year BP**

Methods

The bibliography has been analysed by collating macroremain studies (*sensu lato*) from the Alps. The review only concerns stand-to-local evidences of tree occurrences before the cold climatic ‘8.2-yr BP’ event. Periods before the 8.2-kyr BP event include the Last Glacial Maximum that started to end ca. 18,000 cal yr BP and definitely at 14,500 cal yr BP, the warming Lateglacial from 14,500-11,700 cal yr BP, that includes the cold Late Dryas from 12,900-11,700 cal yr BP, and the warming early-Holocene that ended 8200 yr ago (Clark et al. 2009; Walker et al. 2009). These evidences are based on identification of plant macroremains from lake and peat sediments, plant imprints and charcoal in travertine (calcareous tufa) and radiocarbon dated charcoal from soil (namely pedoanthracology). All these botanical approaches provide accurate identification of *Pinus cembra* at the species level. According to our aim to highlight stand-to-local evidences of *P. cembra*, other classic plant proxies such as pollen or stomata could not be easily used. Indeed, stomata of *P. cembra* are not different from other pine stomata. The problem with pollen was the large uncertainties on the source area of *Pinus* pollen. Further, microscopic identification of *Pinus cembra* (haploxylon pine) from the other *Pinus* (diploxylon) is not based on a clear morphological character excepted based on scanning electronic microscopy, which is not a routine technique in pollen analysis and applied only once in the Alps (Nakagawa et al. 2000); besides, some pollen analysts do not make the distinction between haploxylon and diploxylon pines, unfortunately (e.g. David 1995; Ortu et al. 2008). Finally, pines pollen data still remains indicative of regional trajectories (e.g. Vescovi et al. 2007), but cannot be used to prove their stand-to-local presence as expected here.

The dates of the first occurrence are based on the date indicated in the articles by their authors (Table S1) or sometime reanalysed based on recent calibration progresses of ^14^C measurements. Most dates are inferred from ^14^C or Th/U measurements made on sediment profiles (bulk, total organic carbon, assemblage of plant remains). Some dates result from extrapolation or from palynological chrono-stratigraphy and are thus indicated ‘relative dating’. Soil charcoal dates are direct measurement of the botanically identified charred particles. These dates from the western Alps (Table S1) were completed by other evidences outside the study area and coming from the eastern part of the Alps or northern Italy (east of 8ºE; Table S2).

Table S1: Date of first occurrences of *Pinus cembra* in the western Alps (west of 8ºE) based on analyses of plant macroremains (*sensu lato*) in lake/peat sediment or imprints in travertine. Site order was organised from northeast to the southwest. These dates were used in the Figure 1(c). A = Austria; CH = Switzerland; F = France; I = Italy

| Site name | Alt (m asl) | Lat. / Long. | Dating | Cal yr BP | Bio-proxy | Ref |
| --- | --- | --- | --- | --- | --- | --- |
| Gouillé Rouillon (CH) | 2343 | 46º09′25′′N/07º21′45′′E | 14C | 10,700-10,500 | Macroremains | Tinner et al. 1996;  Tinner & Kaltenrieder 2005 |
| Gouillé Loéré (CH) | 2505 | 46º08’44’N/7º21’33’’E | 14C | ~10,000 | Macroremain, Charcoal | Tinner & Theurillat 2003 |
| Lac du Lait (F) | 2180 | 45°18º52″N/06°48º55″E | 14C | 10,200 | Macroremains | Carcaillet et al 2009 |
| Lac du Loup (F) | 2035 | 45°11’15"N/06°32’16"E | 14C | 11,700 | Macroremains | Blarquez et al 2010 |
| Lac Canard (F) | 2055 | 45º03’42’’N/05º55’49’’E | Relative dating | 8800-8500 | Macroremains | Ponel et al. 1992 |
| Selle (I) | 1890 | 45º04’N/6º55’E | Th/U | 10,150 | Imprints | Ali et al 2006 |
| Jalavez forest (F) | 2100 | 44º39’28’’N/06º48’00’’E | 14C | 9025-8700 | Charcoal | Saulnier et al. 2015 |
| Aigue Agnelle (F) | 2280 | 44º44’N/06º53’E | 14C | 9330-9090 | Charcoal | Ali et al. 2005 |
| Lac Miroir (F) | 2214 | 44°38’03″N/06°47’31″E | 14C | 21,000 | Macroremains | Carcaillet & Blarquez 2007 |

Table S2: Other dates of first occurrences of *Pinus cembra* in the eastern Alps in northern Italy (east of 8ºE) based on analyses of plant macroremain (*sensu lato*) in lake/peat sediment. Site order was organised from east to the west. These dates were not used in the article.

| Site name | Alt (m asl) | Lat. / Long. | Dating | Cal yr BP | Bio-proxy | Ref |
| --- | --- | --- | --- | --- | --- | --- |
| Hirschlich (A) | 2132 | 46°54'16"N/12°15'37"E | 14C | ~11,400 | Macroremains | Oeggl & Wahlmüller 1994 |
| L. di Colbricone Inferiore (I) | 1914 | 46º17’01’’N/11º45’56’’E | 14C | 11,030 | Macroremains | Leys et al 2014 |
| Lej da San Murezzan (CH) | 1768 | 46º29’N/09º50’E | 14C | 10,400 | Macroremains | Gobet et al 2005 |
| Lej da Champfèr (CH) | 1791 | 46°28ºN/09º49’E | 14C | 10,500 | Macroremains | Gobet et al 2005 |
| Lago Basso (I) | 2250 | 46º29’N/09º19’E | 14C | 11,100 | Macroremains | Wick & Tinner 1997 |
| Lago di Ganna (I) | 452 | 45º53’50’’N/8º49’33’’E | Relative dating | 16,000-14,000 | Macroremains | Schneider & Tobolski 1985 |
| Balladrum (CH) | 390 | 46º01’N/8º45’E | 14C | 16,500-14,250 | Macroremains | Hofstetter et al 2006 |
| Simplon-Hobschensee (CH) | 2017 | 46°15'09"N/08°01'25"E | 14C | 11,400-10,900 | Macroremains | Lang & Tobolski 1994 |

**Genetic differentiation**

Table S3: Matrix of pairwise *F*_ST_ values between central and marginal *Pinus cembra* populations in the western Alps.

| ***F*_ST_** | **CAL** | **CAU** | **CAY** | **CBL** | **CLP** | **CLV** | **CMI** | **COR** | **CPL** | **CTU** | **CSC** | **MAR** | **MAU** | **MBR** | **MCH** | **MDE** | **MFL** | **MGI** | **MMO** | **MRO** | **MTA** | **MVA** |
| --- | --- | --- | --- | --- | --- | --- | --- | --- | --- | --- | --- | --- | --- | --- | --- | --- | --- | --- | --- | --- | --- | --- |
| **CAL** | 0.0000 |  |  |  |  |  |  |  |  |  |  |  |  |  |  |  |  |  |  |  |  |  |
| **CAU** | 0.0207 | 0.0000 |  |  |  |  |  |  |  |  |  |  |  |  |  |  |  |  |  |  |  |  |
| **CAY** | 0.0158 | 0.0292 | 0.0000 |  |  |  |  |  |  |  |  |  |  |  |  |  |  |  |  |  |  |  |
| **CBL** | 0.0323 | 0.0318 | 0.0295 | 0.0000 |  |  |  |  |  |  |  |  |  |  |  |  |  |  |  |  |  |  |
| **CLP** | 0.0271 | 0.0396 | 0.0205 | 0.0275 | 0.0000 |  |  |  |  |  |  |  |  |  |  |  |  |  |  |  |  |  |
| **CLV** | 0.0374 | 0.0459 | 0.0229 | 0.0311 | 0.0084 | 0.0000 |  |  |  |  |  |  |  |  |  |  |  |  |  |  |  |  |
| **CMI** | 0.0155 | 0.0161 | 0.0273 | 0.0465 | 0.0372 | 0.0470 | 0.0000 |  |  |  |  |  |  |  |  |  |  |  |  |  |  |  |
| **COR** | 0.0272 | 0.0343 | 0.0226 | 0.0203 | 0.0138 | 0.0148 | 0.0382 | 0.0000 |  |  |  |  |  |  |  |  |  |  |  |  |  |  |
| **CPL** | 0.0434 | 0.0386 | 0.0360 | 0.0268 | 0.0283 | 0.0186 | 0.0463 | 0.0224 | 0.0000 |  |  |  |  |  |  |  |  |  |  |  |  |  |
| **CTU** | 0.0164 | 0.0261 | 0.0165 | 0.0348 | 0.0198 | 0.0249 | 0.0265 | 0.0248 | 0.0303 | 0.0000 |  |  |  |  |  |  |  |  |  |  |  |  |
| **CSC** | 0.0215 | 0.0197 | 0.0232 | 0.0275 | 0.0218 | 0.0246 | 0.0233 | 0.0156 | 0.0211 | 0.0124 | 0.0000 |  |  |  |  |  |  |  |  |  |  |  |
| **MAR** | 0.0243 | 0.0496 | 0.0213 | 0.0444 | 0.0200 | 0.0240 | 0.0310 | 0.0262 | 0.0439 | 0.0237 | 0.0307 | 0.0000 |  |  |  |  |  |  |  |  |  |  |
| **MAU** | 0.0693 | 0.0796 | 0.0635 | 0.0488 | 0.0525 | 0.0487 | 0.0743 | 0.0409 | 0.0559 | 0.0725 | 0.0618 | 0.0593 | 0.0000 |  |  |  |  |  |  |  |  |  |
| **MBR** | 0.0626 | 0.0724 | 0.0580 | 0.0522 | 0.0390 | 0.0452 | 0.0721 | 0.0437 | 0.0648 | 0.0561 | 0.0509 | 0.0580 | 0.0367 | 0.0000 |  |  |  |  |  |  |  |  |
| **MCH** | 0.0264 | 0.0351 | 0.0154 | 0.0416 | 0.0260 | 0.0289 | 0.0271 | 0.0272 | 0.0450 | 0.0162 | 0.0241 | 0.0229 | 0.0542 | 0.0446 | 0.0000 |  |  |  |  |  |  |  |
| **MDE** | 0.0226 | 0.0178 | 0.0153 | 0.0403 | 0.0309 | 0.0293 | 0.0235 | 0.0266 | 0.0350 | 0.0169 | 0.0200 | 0.0339 | 0.0706 | 0.0674 | 0.0184 | 0.0000 |  |  |  |  |  |  |
| **MFL** | 0.0461 | 0.0190 | 0.0515 | 0.0557 | 0.0640 | 0.0768 | 0.0498 | 0.0588 | 0.0738 | 0.0586 | 0.0453 | 0.0810 | 0.1190 | 0.1103 | 0.0658 | 0.0250 | 0.0000 |  |  |  |  |  |
| **MGI** | 0.0637 | 0.0600 | 0.0452 | 0.0326 | 0.0317 | 0.0233 | 0.0774 | 0.0396 | 0.0271 | 0.0415 | 0.0458 | 0.0543 | 0.0561 | 0.0520 | 0.0547 | 0.0565 | 0.0943 | 0.0000 |  |  |  |  |
| **MMO** | 0.0385 | 0.0571 | 0.0298 | 0.0509 | 0.0165 | 0.0184 | 0.0422 | 0.0381 | 0.0367 | 0.0277 | 0.0388 | 0.0251 | 0.0707 | 0.0625 | 0.0345 | 0.0394 | 0.0763 | 0.0412 | 0.0000 |  |  |  |
| **MRO** | 0.1144 | 0.1171 | 0.0962 | 0.0998 | 0.0709 | 0.0657 | 0.1190 | 0.0928 | 0.0847 | 0.0798 | 0.0816 | 0.0647 | 0.1086 | 0.0943 | 0.0807 | 0.0979 | 0.1777 | 0.0632 | 0.0545 | 0.0000 |  |  |
| **MTA** | 0.0107 | 0.0224 | 0.0166 | 0.0372 | 0.0225 | 0.0291 | 0.0136 | 0.0241 | 0.0392 | 0.0112 | 0.0179 | 0.0188 | 0.0705 | 0.0574 | 0.0143 | 0.0161 | 0.0404 | 0.0547 | 0.0265 | 0.0860 | 0.0000 |  |
| **MVA** | 0.0185 | 0.0226 | 0.0211 | 0.0358 | 0.0327 | 0.0407 | 0.0144 | 0.0305 | 0.0379 | 0.0225 | 0.0236 | 0.0352 | 0.0513 | 0.0521 | 0.0202 | 0.0245 | 0.0467 | 0.0584 | 0.0483 | 0.0955 | 0.0240 | 0.0000 |

**Bioclimatic variables and the associated statistics**

Table S4: Means and standard deviation (SDs) of 19 bioclimatic variables.

| **Variable** | **Description** | **Mean** | **SD** |
| --- | --- | --- | --- |
| Bio 1 | Annual Mean Temperature | 3.177 | 0.980 |
| Bio 2 | Mean Diurnal Range (Mean of monthly (max temp - min temp)) | 7.309 | 0.549 |
| Bio 3 | Isothermality (Bio 2/Bio 7) (* 100) | 2.995 | 0.090 |
| Bio 4 | Temperature Seasonality (standard deviation *100) | 582.036 | 16.921 |
| Bio 5 | Max Temperature of Warmest Month | 16.241 | 1.049 |
| Bio 6 | Min Temperature of Coldest Month | -7.755 | 1.285 |
| Bio 7 | Temperature Annual Range (BIO5-BIO6) | 23.995 | 1.130 |
| Bio 8 | Mean Temperature of Wettest Quarter | -1.123 | 2.869 |
| Bio 9 | Mean Temperature of Driest Quarter | 10.609 | 0.953 |
| Bio 10 | Mean Temperature of Warmest Quarter | 10.686 | 0.961 |
| Bio 11 | Mean Temperature of Coldest Quarter | -3.991 | 1.114 |
| Bio 12 | Annual Precipitation | 1367.909 | 154.152 |
| Bio 13 | Precipitation of Wettest Month | 133.727 | 16.994 |
| Bio 14 | Precipitation of Driest Month | 88.000 | 15.538 |
| Bio 15 | Precipitation Seasonality (Coefficient of Variation) | 11.000 | 2.182 |
| Bio 16 | Precipitation of Wettest Quarter | 379.818 | 43.228 |
| Bio 17 | Precipitation of Driest Quarter | 297.909 | 36.362 |
| Bio 18 | Precipitation of Warmest Quarter | 309.318 | 42.329 |
| Bio 19 | Precipitation of Coldest Quarter | 361.500 | 57.445 |

Table S5: Bioclimatic variables loadings for the first two axes (PC1 and PC2) of the principle component analysis (PCA).

| **ID** | **PC1 (axis 1)** | **PC2 (axis 2)** |
| --- | --- | --- |
| CAL | -1.823445 | -1.6275085 |
| CAU | 2.277648 | -0.05683665 |
| CAY | -0.802847 | 0.32237532 |
| CBL | 3.060321 | 1.44635711 |
| CLP | -0.534912 | 0.34191329 |
| CLV | 0.968391 | 0.23748161 |
| CMI | 0.846951 | -3.65319161 |
| COR | -1.02701 | 4.88704516 |
| CPL | 3.330867 | 0.21787104 |
| CTU | 1.705786 | -1.43262491 |
| CSC | 0.250404 | 3.70986727 |
| MAR | 4.466884 | 0.46278585 |
| MAU | -7.992288 | 0.26943008 |
| MBR | -1.552138 | -3.29211759 |
| MCH | 1.175563 | 0.44553237 |
| MDE | -2.648538 | 1.49519601 |
| MFL | 6.128895 | -1.61350572 |
| MGI | 2.82539 | 1.77399598 |
| MMO | -3.239757 | -1.92735292 |
| MRO | -8.629001 | 0.07170586 |
| MTA | 1.091443 | -0.24148619 |
| MVA | 0.121392 | -1.83693285 |

**Figures**


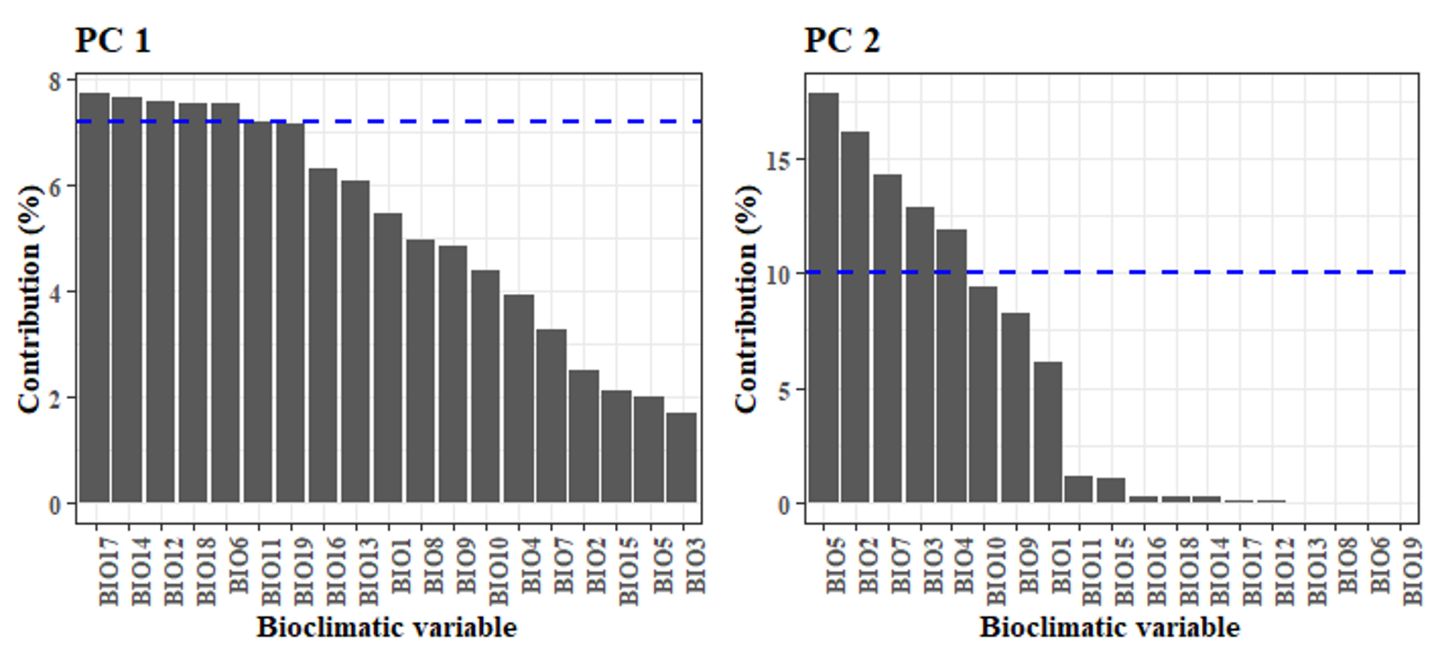


Figure S1: Contribution of bioclimatic variables for the first two axes (PC1 and PC2) of the principle component analysis (PCA). The highest five contributing variables are above the dashed blue line.

**References**

Ali A.A., Carcaillet C., Talon B., Roiron P. & Terral J.-F. (2005) *Pinus cembra* L. (arolla pine). a common tree in the inner French Alps since the early Holocene and above the present tree line: a synthesis based on charcoal data from soils and travertines. *Journal of Biogeography*. 32. 1659-1669

Ali A.A., Martinez M.. Fauvart N., Roiron P., Fioraso G., Guendon J.-L., Terral, J.-F. & Carcaillet C. (2006) Incendies et peuplements à *Pinus mugo* Turra dans les Alpes occidentales (Val de Suse. Italie) durant la transition Tardiglaciaire – Holocène : une zone refuge évidente. *Comptes Rendus Biologies*. 329. 494-501

Blarquez O., Carcaillet C., Mourier B., Bremond L. & Radakovitch O. (2010). Trees in the subalpine belt since 11 700 cal. BP: Origin. expansion and alteration of the modern forest. *The Holocene*. 20. 139–146. <https://doi.org/10.1177/0959683609348857>

Carcaillet C. & Blarquez O. (2017). Fire ecology of a tree glacial refugium on a nunatak with a view on Alpine glaciers. *New Phytologist.* 216. 1281–1290. doi:10.1111/nph.14721

Carcaillet C., Ali A.A., Blarquez O., Genries A., Mourier B. & Bremond. L. (2009). Spatial variability of fire history in subalpine forests: from natural to cultural regimes. *Ecoscience*. 16: 1–12.

Clark P.U., Dyke A.S., Shakun J.D., Carlson A.E., Clark J., Wohlfarth B., Mitrovica J.X., Hostetler S.W. & McCabe A.M. (2009). The last glacial maximum. *Science*. 325. 710–714. https://doi.org/10.1126/science.1172873

David F. (1995). Vegetation dynamics in the northern French Alps. *Historical Biology 9*. 269–95.

Gobe E., Tinner W., Bigler C., Hochuli P.A. & Ammann B. (2005). Early-Holocene afforestation processes in the lower subalpine belt of the Central Swiss Alps as inferred from macrofossil and pollen records. *The Holocene*. 15. 672–686. https://doi.org/10.1191/0959683605hl843rp

Hofstetter S., Tinner W., Valsecchi V., Carraro G. & Conedera M. (2006). Late-glacial and Holocene vegetation history in the Insubrian Southern Alps—new indications from a small-scale site. *Vegetation History and Archaeobotany* 15. 87–98.

Lang G. & Tobolski K. (1985). Hobschensee - Late-Glacial and Holocene environment of a lake near the timberline. Dissertationes Botanicae 87. 209-228.

Leys B., Carcaillet C., Blarquez O., Lami. A., Musazzi S. & Trevisan R. (2014). Resistance of mixed subalpine forest to fire frequency changes: the ecological function of dwarf pine (*Pinus mugo* ssp. *mugo*). *Quaternary Science Reviews* 90. 60-68. DOI: 10.1016/j.quascirev.2014.02.023

Nakagawa T., Edouard J.L. & de Beaulieu J.L. (2000). A scanning electron microscopy (SEM) study of sediments from Lake Cristol. southern French Alps. with special reference to the identification of *Pinus cembra* and other Alpine *Pinus* species based on SEM pollen morphology. *Review of Palaeobotany and Palynology*. 108. 1-15.

Oeggl K. & Wahlmüller N. (1994). Holozäne Vegetationsentwicklung an der Waldgrenze der Ostalpen: Die Plancklacke (2140 m)/Sankt Jakob im Defreggen. Osttirol. *Dissertationes Botanicae* 234. 389-411.

Ortu E., de Beaulieu J.L.. Caramiello R., Siniscalco C. (2008) Late glacial and Holocene vegetation dynamics at various altitudes in the Ellero Valley. Maririme Alps. northwestern Italy. *Ecoscience* 15. 200-212.

Ponel P. de Beaulieu J.L & Tobolski K. (1992) Holocene palaeoenvironments at the timberline in the Taillefer Massif. French Alps: a study of pollen. plant macrofossils and fossil insects. *The Holocene* 2(2). 117-130

Schneider R. & Tobolski K. (1985) Lago di Ganna – Lateglacial and Holocene environments of a lake in the southern Alps. *Dissertationes Botanicae* 87. 229–271

Tinner W., Ammann. B. & Germann P. (1996). Treeline fluctuations recorded for 12 500 years by soil profiles. pollen. and plant macrofossils in the central Swiss Alps. *Arctic and Alpine Research* 28. 131-147.

Tinner W. & Kaltenrieder P. (2005). Rapid response of high mountain vegetation to early Holocene environmental changes in the Swiss Alps. *Journal of Ecology* 93. 936–47.

Tinner W. & Theurillat J.P. (2003). Uppermost limit. extent. and fluctuations of the timberline and treeline ecocline in the Swiss Central Alps during the past 11 500 years. *Arctic Antarctic and Alpine Research* 35. 158-169.

Vescovi E., Ravazzi C., Arpenti E., Finsinger W., Pini R., Valsecchi V., Wick L., Ammann B. & Tinner W. (2007). Interactions between climate and vegetation during the Lateglacial period as recorded by lake and mire sediment archives in Northern Italy and Southern Switzerland. *Quaternary Science Reviews* 26(11–12). 1650–1669.

Walker M. Johnsen S. Rasmussen S.O., Popp T., Steffensen J. P., Gibbard P., Hoek W., Lowe J., Andrews J., Björk S., Cwynar LC., Hughen K., Kershaw P., Kromer B., Litt T., Lowe D.J., Nakagawa T., Newnham R. & Schwander J. (2009). Formal definition and dating of the GSSP (Global Stratotype Section and Point) for the base of the Holocene using the Greenland NGRIP ice core. and selected auxiliary records. *Journal of Quaternary Science*. 24. 3–17.

Wick L. & Tinner W. (1997). Vegetation changes and timberline fluctuations in the Central Alps as indicators of Holocene climatic oscillations. *Arctic and Alpine Research* 29. 445-458
